# The effect of dietary interventions on pain and quality of life in women diagnosed with endometriosis: a prospective study with control group

**DOI:** 10.1093/humrep/dead214

**Published:** 2023-10-24

**Authors:** A P van Haaps, J V Wijbers, A M F Schreurs, S Vlek, J Tuynman, B De Bie, A L de Vogel, M van Wely, V Mijatovic

**Affiliations:** Department of Reproductive Medicine, Amsterdam University Medical Centers, Vrije Universiteit, Amsterdam, The Netherlands; Endometriosis Center, Amsterdam University Medical Centers, Academic Medical Center, Amsterdam, The Netherlands; Amsterdam Reproduction and Development Research Institute, Amsterdam, The Netherlands; Endometriosis Center, Amsterdam University Medical Centers, Academic Medical Center, Amsterdam, The Netherlands; Faculty of Health, Nutrition & Sport, The Hague University of Applied Sciences, Den Haag, The Netherlands; Department of Reproductive Medicine, Amsterdam University Medical Centers, Vrije Universiteit, Amsterdam, The Netherlands; Endometriosis Center, Amsterdam University Medical Centers, Academic Medical Center, Amsterdam, The Netherlands; Amsterdam Reproduction and Development Research Institute, Amsterdam, The Netherlands; Endometriosis Center, Amsterdam University Medical Centers, Academic Medical Center, Amsterdam, The Netherlands; Department of Surgery, Amsterdam University Medical Centers, Vrije Universiteit, Amsterdam, The Netherlands; Endometriosis Center, Amsterdam University Medical Centers, Academic Medical Center, Amsterdam, The Netherlands; Department of Surgery, Amsterdam University Medical Centers, Vrije Universiteit, Amsterdam, The Netherlands; Endometriose Stichting, Dutch Patient Organization for Endometriosis, Sittard, The Netherlands; Dietician Practice Aileen de Vogel, Dordrecht, The Netherlands; Department of Reproductive Medicine, Amsterdam University Medical Centers, Academic Medical Center, Amsterdam, The Netherlands; Department of Reproductive Medicine, Amsterdam University Medical Centers, Vrije Universiteit, Amsterdam, The Netherlands; Endometriosis Center, Amsterdam University Medical Centers, Academic Medical Center, Amsterdam, The Netherlands; Amsterdam Reproduction and Development Research Institute, Amsterdam, The Netherlands

**Keywords:** dietary intervention, self-management, endometriosis, low FODMAP diet, low fermentable oligo-, di-, mono-saccharides, and polyols, endometriosis diet, pain, quality of life

## Abstract

**STUDY QUESTION:**

What is the influence of dietary interventions, namely the low fermentable oligo-, di-, mono-saccharides, and polyols (Low FODMAP) diet and endometriosis diet, on endometriosis-related pain and quality of life (QoL) compared to a control group?

**SUMMARY ANSWER:**

After adhering to a dietary intervention for 6 months, women with endometriosis reported less pain and an improved QoL compared to baseline whereas, compared to the control group, they reported less bloating and a better QoL in 3 of 11 domains.

**WHAT IS KNOWN ALREADY:**

Standard endometriosis treatment can be insufficient or may be accompanied by unacceptable side effects. This has resulted in an increasing interest in self-management strategies, including the appliance of the Low FODMAP diet and the endometriosis diet (an experience-based avoidance diet, developed by women with endometriosis). The Low FODMAP diet has previously been found effective in reducing endometriosis-related pain symptoms, whereas only limited studies are available on the efficacy of the endometriosis diet. A survey study recently found the endometriosis diet effective in improving QoL but currently no guidelines on use of the diet exist.

**STUDY DESIGN, SIZE, DURATION:**

A prospective one-center pilot study was performed between April 2021 and December 2022. Participants could choose between adherence to a diet—the Low FODMAP diet or endometriosis diet—or no diet (control group). Women adhering to a diet received extensive guidance from a dietician in training. The follow-up period was 6 months for all three groups. For all outcomes, women adhering to the diets were compared to their baseline situation and to the control group.

**PARTICIPANTS/MATERIALS, SETTING, METHODS:**

We included women diagnosed with endometriosis (surgically and/or by radiologic imaging) who reported pain scores ≥3 cm on the visual analogue score (0–10 cm) for dysmenorrhea, deep dyspareunia, and/or chronic pelvic pain. The primary endpoint focused on pain reduction for all pain symptoms, including dysuria, bloating, and tiredness. Secondary endpoints, assessed via questionnaires, focused on QoL, gastro-intestinal health, and diet adherence.

**MAIN RESULTS AND THE ROLE OF CHANCE:**

A total of 62 participants were included in the low FODMAP diet (n = 22), endometriosis diet (n = 21), and control group (n = 19). Compared to their baseline pain scores, participants adhering to a diet reported less pain in four of six symptoms (range *P* < 0.001 to *P* = 0.012) and better scores in 6 of 11 QoL domains (range *P* < 0.001 to *P* = 0.023) after 6 months. Compared to the control group, analyzed longitudinally over the 6-month follow-up period, participants applying a diet reported significant less *bloating* (*P* = 0.049), and better scores in 3 of 11 QoL domains (range *P* = 0.002 to *P* = 0.035).

**LIMITATIONS, REASONS FOR CAUTION:**

No sample size was calculated since efficacy data were lacking in the literature. In order to optimize dietary adherence, randomization was not applied, possibly resulting in selection bias.

**WIDER IMPLICATIONS OF THE FINDINGS:**

Our study suggests that women could benefit from adherence to a dietary intervention, since we found lower pain scores and better QoL after 6 months. However, caution is implied since this is a pilot study, no sample size was calculated, and data on long-term effects (>6 months) are lacking. The results of this pilot study underline the importance of further research and the drawing up of guidelines.

**STUDY FUNDING/COMPETING INTEREST(S):**

A.v.H. reports receiving a travel grant from Merck outside the scope of this study. J.W., S.V., J.T., and B.D.B. have no conflicts of interest to report. A.d.V. reports having received KP-register points for internship guidance of J.W., performing paid consultations with endometriosis patients outside the study and receiving reimbursements for educational lectures at the local hospital (Albert Schweitzer Ziekenhuis, Dordrecht, the Netherlands). A.S. reports having received expenses for travel and hotel costs as an invited speaker from ESHRE. This was outside the scope of this study. M.v.W. reports that she is a Co-Ed of *Cochrane Gynecology and Fertility*. V.M. reports receiving travel and speaker’s fees from Guerbet and research grants from Guerbet, Merck and Ferring. The department of reproductive medicine (V.M.) of the Amsterdam UMC, location VUmc, has received several research and educational grants from Guerbet, Merck and Ferring not related to the submitted work.

**TRIAL REGISTRATION NUMBER:**

N/A.

## Introduction

Endometriosis is characterized by the presence of endometrium-like tissue outside the uterus, and can be described as an estrogen-dependent, chronic inflammatory systemic condition (Becker *et al.*, 2022). Endometriosis affects ∼10% of women of reproductive age worldwide. Symptoms associated with endometriosis can be cyclical, including dysmenorrhea, dyschezia and dysuria, and non-cyclical, including chronic pelvic pain, deep dyspareunia and infertility (Zondervan *et al.*, 2020; Becker *et al.*, 2022). Other symptoms occurring with endometriosis, such as bloating, abdominal cramping, back pain and fatigue, may overlap with chronic pain conditions such as irritable bowel syndrome (IBS). This overlap in addition to the lack of endometriosis-specific symptoms and available biomarkers may contribute to the current diagnostic delay of 3–11 years (Dunselman *et al.*, 2014; Moore *et al.*, 2017; Ciebiera *et al.*, 2021).

Treatment options for endometriosis include hormonal therapy, surgery, pain management, and/or treatment with ART (Becker *et al.*, 2022). However, these medical options can be insufficient in treating symptoms or may be accompanied with unacceptable side effects. Surgery may be accompanied by complications and its effect may diminish in time, leading to recurrence of endometriosis lesions after surgery, especially in hormonally untreated patients. The recurrence of endometriosis lesions often results in the recurrence of symptoms (Nirgianakis *et al.*, 2020; Gutke *et al.*, 2021; Saunders and Horne, 2021). Previous research found that endometriosis and the associated symptoms can negatively affect both mental and physical quality of life (QoL) (Culley *et al.*, 2013; De Graaff *et al.*, 2013; Della Corte *et al.*, 2020). Therefore, there has been an increasing interest among women with endometriosis in the application of self-management strategies such as meditation, breathing exercises, acupuncture, the use of cannabis—or Cannabidiol (CBD) oil—and dietary interventions (Armour *et al.*, 2019). The appliance of self-management strategies was recently found to be associated with a positive effect on physical and mental QoL (O'Hara *et al.*, 2021).

In recent years, several studies have been performed on the effect of an adjustment in nutrient intake on endometriosis-related symptoms and QoL. According to a recent systematic review, the addition of certain nutrients (e.g. omega-3 and -6, several vitamins and minerals) and the avoidance of other nutrients (e.g. gluten and soy) had a positive effect on endometriosis-related symptoms (Huijs and Nap, 2020). This finding was confirmed by a study that found that supplements, such as omega-3, had a positive effect on endometriosis-associated pain (Yalcin Bahat *et al.*, 2022). Studies on the effectiveness of dietary interventions commonly used by women with endometriosis (i.e. a gluten free or lactose free diet) are limited. Nevertheless, in these limited studies the dietary interventions have been found effective in managing endometriosis-related symptoms. However, the mechanisms of action involved are largely unknown (Marziali *et al.*, 2012; Moore *et al.*, 2017; Bellini *et al.*, 2020; Vennberg Karlsson *et al.*, 2020).

The low fermentable oligo-, di-, mono-saccharides, and polyols (Low FODMAP) diet, developed by Australian researchers a decade ago, has been studied frequently. It is considered the most effective diet for treating IBS-related symptoms (Staudacher and Whelan, 2017). Monash University has developed a guideline on the implementation of the Low FODMAP diet (Gibson and Shepherd, 2010; Monash, 2019). Additionally, the study by Moore *et al.* (2017) suggested a specific effect of the Low FODMAP diet on endometriosis-related symptoms. They found that the Low FODMAP diet was significantly more effective in reducing pain symptoms in women diagnosed with both IBS and endometriosis compared to women diagnosed with IBS alone (Moore *et al.*, 2017; Bellini *et al.*, 2020).

In contrast to the Low FODMAP diet, the endometriosis diet is an experience-based diet developed and frequently applied by women with endometriosis in the Netherlands. Evidence regarding the efficacy of the endometriosis diet is very limited and currently no guidelines on the use, or the exact composition, of the endometriosis diet exist (Muntslag *et al.*, 2020; Krabbenborg *et al.*, 2021; van Haaps *et al.*, 2023). In addition, there are indications that the consumption of some nutrients, such as gluten or dairy, which are avoided when applying the endometriosis diet, could be beneficial for the composition of the gut microbiome and are part of a fully fledged diet. Finally, data regarding possible negative effects of these diets owing to the removal of essential nutrients and its effect on health are lacking. Therefore, independently applying the endometriosis diet currently is not recommended by dieticians. Dietary guidance is needed to ensure a fully fledged diet and one that is based on the patients’ symptom pattern (Huijs and Nap, 2020; Schwartz *et al.*, 2022; Brouns, 2023). However, a recently published survey found that adherence to the endometriosis diet was associated with a significantly higher QoL among women with endometriosis, when compared to non-diet adherence. Strict adherence to the diet showed higher scores in all QoL domains when compared to less strict adherence (van Haaps *et al.*, 2023). Non-medical interventions, such as dietary adjustments and interventions, are discussed in the 2022 ESHRE guideline on the management of endometriosis (Becker *et al.*, 2022). It is recommended to discuss the application of a dietary intervention with the patient. However, since data on long-term effects and possible harmful effects are lacking, no specific recommendations are made on the appliance of a dietary intervention in women with endometriosis (Becker *et al.*, 2022). It is therefore currently not possible to provide a sufficient scientifically substantiated answer to the question frequently asked in daily practice by women diagnosed with endometriosis, of whether they should apply a dietary intervention for their symptoms.

Therefore, the objective of this study was to broaden our knowledge and determine the impact of two dietary interventions, namely the Low FODMAP diet and the endometriosis diet, on endometriosis-related (pain) symptoms and QoL. The two diets are commonly used by women with endometriosis. The aim was to evaluate their capacity to reduce pain symptoms and improve QoL, as well as to identify facilitators and barriers of dietary adjustments. The two dietary interventions were compared to a control group of women not adhering to a diet. To the best of our knowledge, this approach not yet been applied in previous studies.

## Materials and methods

This prospective pilot study was performed at the Endometriosis Center of the Amsterdam University Medical Center, Amsterdam, the Netherlands. It was conducted between April 2021 and December 2022. The study was approved by the institutional review board of the Amsterdam UMC and was retrospectively registered at www.clinicaltrials.gov (reference number NCT05714189), because of the temporary hiatus between the closure of the Dutch Trial Register and the possibility to register them at www.clinicaltrials.gov.

Women screened for participation were all diagnosed with endometriosis, either through radiologic imaging (transvaginal ultrasound and/or MRI) or laparoscopy. Women were eligible for participation when they experienced insufficient benefit from their current medical treatment, with a reported pain score of ≥3 (visual analogue score (VAS), scale 0–10 cm) in one or more of the following symptoms: dysmenorrhea, deep dyspareunia, and chronic pelvic pain. Women were excluded when they had undergone surgery in the 6 weeks prior to study enrollment, when they were to undergo surgery within the next 6 months, or when a switch in hormonal therapy within 6 weeks was imminent. In addition, women were excluded if they were pregnant, breastfeeding, diagnosed with a malignancy, or if they were additionally diagnosed with irritable bowel disease and/or lactose intolerance. Finally, women had to be sufficiently fluent in the Dutch or English language.

As this was a pilot study, we aimed to include 20 participants per group, resulting in a total of 60 study participants. During the inclusion period, seven women were lost to follow up after they signed their informed consent but prior to them starting a diet or adherence to the control group. Furthermore, one participant decided to withdraw from the study after adhering to her chosen dietary intervention for 3 weeks. Two participants withdrew from study participation after adhering to their chosen dietary intervention for 6 weeks. We therefore continued inclusion after we reached 60 participants, aiming to include 20 participants per group. Replacements were sought and found for the participants that withdrew from study participation, resulting in a total study population of 62 patients available for analysis.

### The dietary interventions

To optimize diet adherence, participants were able to choose between the dietary interventions (the Low FODMAP diet or the endometriosis diet or the control group) rather than being randomized. When adhering to one of the two dietary interventions, participants were extensively guided by a dietician in training over a period of 3 months. Guidance from a dietician when applying a dietary intervention is encouraged because the independent avoidance by patients of (unnecessary) nutrients and/or certain foods can lead to an incomplete diet. The dietician in training was supervised by a dietician registered in the Paramedics Quality Register. Subsequently participants were asked to continue adherence to the diet without guidance for another 3 months.

The dietary guidance was based on the Dutch dietary guideline for Diverticular Disease and IBS and the Dutch dietary guideline for Inflammatory Bowel Diseases (van der Marel-Sluijter, 2009; van Dijk and Helfrich, 2019). Every participant received the same dietary guidance during the course of the study. A fixed structure of guidance for both dietary interventions was initiated in accordance with the six steps of methodical action (Leibbrandt, 2016). The dietary guidance for both dietary interventions consisted of three 1-h consultations and three short 30-min consultations. Before each initial consultation, participants were asked to complete a food diary for 3 days to monitor their eating behavior. For the Low FODMAP diet, participants had two consultations for each phase: the elimination phase, reintroduction phase, and personalization phase. Since no dietary guideline currently exists for the endometriosis diet, the dietary treatment design of the Low FODMAP diet was also applied for the endometriosis diet. The dietary guidance was concluded with a final interview at the end of the study, where advice for the long term was given if participants decided they wanted to continue their chosen dietary intervention.

When participants decided they did not want to adhere to a dietary intervention, they could be part of the control group. The control group received care as usual without a dietary intervention or guidance by a dietician in training. There was no cross-over between the two dietary interventions and the no-diet group.

To support participants in this study that chose to adhere to a diet, tools were distributed to optimize their diet adherence. For participants adhering to the endometriosis diet, specially developed materials, mostly consisting of suitable recipes, were supplied. For participants adhering to the Low FODMAP diet, specially developed materials, consisting of weekly menus, checklists regarding nutrients or the amount that women could or could not eat and grocery lists, were supplied. Finally, the dietician in training developed a food guide containing practical tips and information making adherence to both dietary interventions easier.

All participants were asked to complete three sets of questionnaires over a period of 6 months. They were distributed at the start of the study (T0), at 3 months follow-up (T1), and at 6 months follow-up, at the end of the study (T2). All three sets of questionnaires contained questions on the participants’ pain scores, expressed using the VAS (scale 0–10 cm) for the symptoms dysmenorrhea, deep dyspareunia, chronic pelvic pain, dysuria, tiredness, and bloating. In addition, the Gastro-Intestinal Quality of Life Index (GIQLI) questionnaire, used to calculate gastro-intestinal health, was included in all three sets of questionnaires. The GIQLI score ranges from 0 to 144, where 0 represents the worst possible gastro-intestinal health and 144 represents the best possible gastro-intestinal health. Finally, all sets of questionnaires contained a questionnaire to specify QoL: the Endometriosis Health Profile (EHP-30) questionnaire. The EHP-30 is validated for use in the Dutch language (Jones *et al.*, 2001; van de Burgt *et al.*, 2011). Using the EHP-30, different scores can be calculated for five core QoL domains (pain, powerlessness, emotional wellbeing, social support, self-image) and six modular QoL domains (work life, children, sexual intercourse, medical profession, treatment, and infertility). The QoL scores range from 0 to 100, where 0 represents best possible health status and 100 the worst possible health status.

For all groups, the first set of questionnaires, distributed at the start of the study (T0), contained a questionnaire measuring basic demographics (e.g. length, weight), the participants’ level of education, the Bristol Stool chart (Lewis and Heaton, 1997) and whether the participant had ever adhered to a diet before. When adhering to a dietary intervention, the second and third set of questionnaires, distributed at 3 months (T1) and at 6 months follow-up (T2), respectively, contained a questionnaire with self-composed questions on the participants’ self-reported strictness score (in VAS) and adherence to their chosen diet. Finally, the third set of questionnaires distributed at 6 months follow-up (T2) contained a questionnaire for the dietary intervention group only measuring the participants satisfaction with the guidance provided by the dietician in training.

### The low FODMAP diet

FODMAPs are a large class of small non-digestible carbohydrates, which can be found in all kinds of nutrients such as fruits, vegetables, honey, sweeteners, and milk and dairy products. Because of their osmotic activity, FODMAPs force water into the gastro-intestinal tract, resulting in the presentation of a food that is easy for the intestinal microbiota in the large intestine to use. This in turn results in fermentation and increased gas production in the large intestine causing luminal distention. All this can result in symptoms such as bloating, flatulence, abdominal pain, and constipation (Gibson and Shepherd, 2010; Monash, 2019; Bellini *et al.*, 2020). The Low FODMAP diet was initially developed for patients diagnosed with IBS. It is currently recommended by the American College of Gastroenterology for the improvement of IBS symptoms, especially in patients that see a link between food, eating, and their IBS symptoms (Gibson and Shepherd, 2010; Monash, 2019). When applying the Low FODMAP diet, IBS symptoms, such as bloating and abdominal pain, are the most likely to improve but one may additionally see improvements in fatigue, bowel movements, and QoL as well (Staudacher and Whelan, 2017; Monash, 2019; Bellini *et al.*, 2020).

The Low FODMAP diet is an avoidance diet and consists of three phases. In the first phase, all nutrients high in FODMAPs (high-FODMAPS) are eliminated from the daily diet over a period of 6–10 weeks to calm down the bowel. After all IBS symptoms are reduced or have even disappeared, patients can continue to the second phase where FODMAP challenges are added. The patient continues the Low FODMAP diet but reintroduces one high-FODMAP nutrient once every 3 days to see whether this exposure causes IBS symptoms. When it does not cause any symptoms, the patient can continue eating this high-FODMAP group in their future daily diet. After each FODMAP challenge, patients will avoid all high-FODMAPS again. Patients can continue to the third and final phase when all FODMAP-challenges are tested. During this phase, the diet is fully personalized and is based on whether the patient tolerated the high-FODMAP nutrient or not during the FODMAP challenges. Only when the high-FODMAP nutrient was not tolerated is it advised to permanently remove it from the daily diet. The Monash University has developed several apps and cookbooks to support women who adhere to the Low FODMAP diet. Our study participants were advised to use them during the course of the study (Monash, 2019).

### The endometriosis diet

The endometriosis diet is an avoidance diet developed by women with endometriosis (www.endometriose.nl, Endometriosestichting, 2023). With the endometriosis diet, women avoid nutrients they noticed provoked or aggravated their endometriosis-related symptoms. For this study, we standardized the diet. We based the contents of the endometriosis diet on a recent survey among Dutch patients with endometriosis where the exact content of the diet was described and its influence on QoL was studied (van Haaps *et al.*, 2023). The endometriosis diet and its composition is described in Table 1. During dietary guidance it was ensured that the participants had a fully fledged diet, despite the fact that certain nutrients were avoided as part of the endometriosis diet.

**Table 1.** Nutrients and nutrient groups to be avoided in the endometriosis diet.

Nutrient groups	Examples
Red meat	Beef, pork, lamb, mutton, veal, horse, liver
Gluten	(Khorasan) wheat, rye, spelt, barley
Cow milk	A sugar found in, e.g. milk, cheese spreads, whey-based soft drinks, ice cream
Sugars	All added sugars. Refined sugars (cane sugar, caster sugar), natural sugars (honey, palm sugar, maple syrup, coconut blossom sugar), and sweeteners (aspartame, cyclamate, saccharin, sucralose, polyols)
Nutrients high in estrogen	Soy (soymilk, tofu, soya sprout, miso, tempeh, soy sauce, natto), linseed, sesame seeds, black beans
Limited caffeine (max 200 mg daily)	Coffee, tea (black tea, green tea, white tea), soft drinks (coca cola, iced tea, energy drinks/shots, chocolate milk), other (dark and/or milk chocolate, pain killer with added caffeine)

### Outcomes

The primary outcome focused on pain scores (the VAS, scale 0–10 cm) of the endometriosis-related symptoms dysmenorrhea, deep dyspareunia, chronic pelvic pain, dysuria, tiredness, and bloating. The secondary outcomes focused on QoL (EHP-30 questionnaire), gastro-intestinal health (GIQLI questionnaire), and adhesion to the dietary intervention. For all outcomes, women adhering to the diets were compared to their baseline situation and to the control group.

### Data collection and analysis

Data were collected using Castor EDC (Castor Electronic Data Capture, Ciwit BV, Amsterdam, the Netherlands) and analyzed using IBM SPSS for Windows, version 26.0 (IBM Corp., Armonk, NY, USA). Because replacement was sought for the participants that withdrew from participation 3–6 weeks after starting their dietary intervention, data were analyzed according to the per-protocol principle. To optimize result liability, data were additionally analyzed according to the intention-to-treat principle. Baseline characteristics were presented as continuous variables using descriptive statistics with mean and SD for normally distributed data and median with interquartile ranges (IQR) for non-normally distributed data. Categorical variables were presented as absolute numbers and percentages. Differences between baseline characteristics and pain scores between baseline and 6-month follow-up were calculated using the independent Student’s *t*-test when there was normally distributed data, and the Mann–Whitney *U* test when there was non-normally distributed data. Differences between the control group and intervention group, analyzed longitudinally over the course of the 6-month follow-up period, were calculated using linear mixed models analysis. The influence of self-reported strictness on pain scores was calculated using a logistic regression. Two-sided *P*-values of <0.05 were considered to indicate statistical significance. Since this was a pilot study, no sample size calculation was performed.

## Results

**Figure 1.**
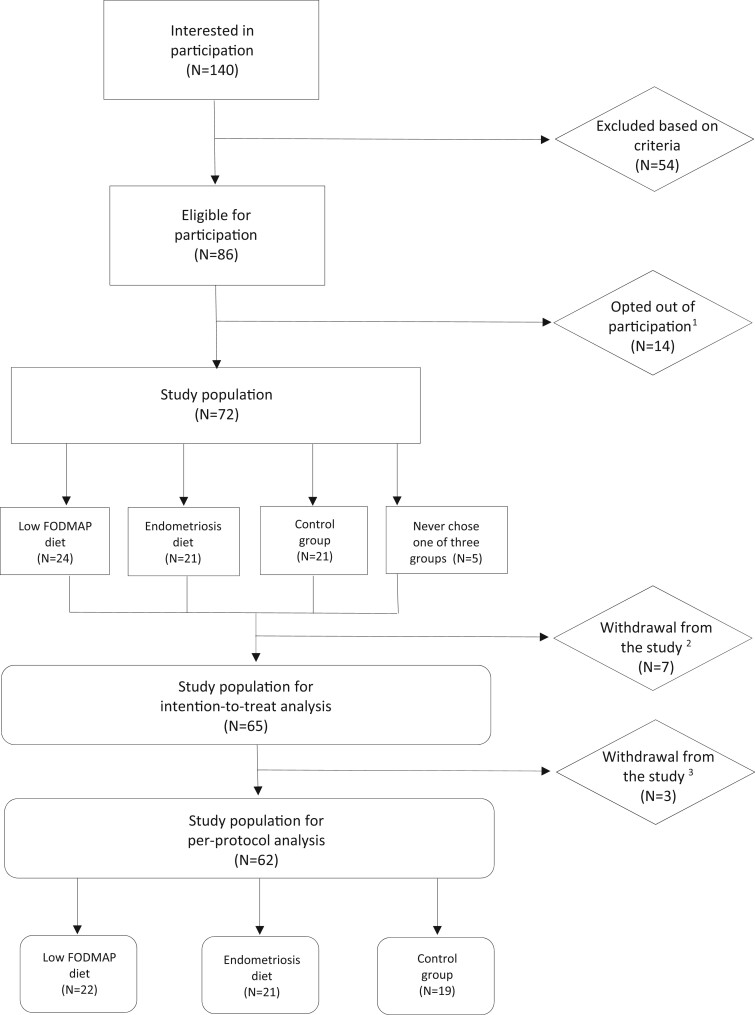
**Flowchart of patient recruitment and inclusion in a prospective study of the effect of diet on pain and quality of life in women with endometriosis.** (1) Opted out of participation because they already started the diet somewhere else, found the diet too drastic or we never heard from them again. (2) FODMAP: fermentable oligo-, di-, mono-saccharides, and polyols. (3) Number of women that withdrew from the study after signing their permission form, but prior to starting their diet or adherence to the control group. (4) Withdrawal from the study after adhering to the diet either for 3 or 6 weeks.

Between April 2021 and August 2022, 140 women indicated they were interested in study participation. Ultimately, 62 women were included in the analysis (Fig. 1).

A total of 43 participants chose to adhere to a diet, of which 22 participants chose to adhere to the Low FODMAP diet and 21 participants chose to adhere to the endometriosis diet. A total of 19 participants decided not to adhere to any diet, and therefore be part of the control group. All patients’ characteristics are presented in Table 2. No statistically significant differences were seen in baseline characteristics between diet users and controls (Table 2).

**Table 2.** Patient characteristics of all three groups, including women adhering to the Low FODMAP diet, endometriosis diet, or no diet (control group).

Patient characteristics	Low FODMAP diet^1^ (n = 22)	Endometriosis diet (n = 21)	Control group (n = 19)	*P*-value
**Age in years (mean (SD))**	36.9 (5.9)	39.1 (15.8)^2^	37.6 (8.5)	0.593
**BMI kg/m^2^** **(mean (SD))**	26.0 (4.4)	24.3 (3.3)	25.4 (4.4)	0.490
**Lifestyle (%)**				
Smoking	1 (4.5%)	0 (0%)	3 (15.8%)	
Alcohol usage	16 (72.7%)	8 (38.1%)	9 (47.4%)	
**Family composition (%)**				
Single	6 (27.3%)	1 (4.8%)	2 (10.5%)	
Living with housemate, partner and/or children	14 (63.6%)	20 (95.2%)	15 (78.9%)	
Unknown	2 (9.1%)	0 (0%)	2 (10.5%)	
**Educational attainment (%)**				
High school	1 (4.5%)	0 (0%)	0 (0%)	
Intermediate or higher vocational education	9 (40.9%)	15 (71.4%)	15 (78.9%)	
University	10 (45.5%)	7 (33.3%)	3 (15.8%)	
Other	1 (4.5%)	0 (0%)	0 (0%)	
Unknown	1 (4.5%)	0 (0%)	1 (5.3%)	
**Other conditions**				
Gynecological diagnoses^3^	–	3 (14.3%)	2 (10.5%)	
Gastro-intestinal, liver, and urinary diagnoses^4^	2 (9.1%)	2 (9.5%)	4 (21.1%)	
Metabolic disorders^5^	–	–	1 (5.3%)	
Neurological diagnoses^6^	1 (4.5%)	2 (9.5%)	1 (5.3%)	
Endocrine disorders^7^	1 (4.5%)	1 (4.8%)	–	
Hemophilia or cardiovascular disease^8^	1 (4.5%)	2 (9.5%)	–	
Muscle diagnoses (fibromyalgia)	2 (9.1%)	1 (4.8%)	1 (5.3%)	
Atopic diagnoses^9^	2 (9.1%)	3 (14.3%)	1 (5.3%)	
Viral diagnoses (cytomegalovirus, covid)	2 (9.1%)	–	1 (5.3%)	
Food allergies/sensitivities, vitamin shortage	–	3 (14.3%)	1 (5.3%)	
Previous malignancy	–	–	1 (5.3%)	
Psychological diagnoses^10^	3 (13.6%)	3 (14.3%)	5 (26.3%)	
**Years since diagnosis (mean, range)**	9.3 (2–20 years)	10.3 (2–35 years)	9.7 (2–26 years)	0.417
**Grade of endometriosis according to ASRM1^1^ (%)**				
Grade I	0 (0%)	0 (0%)	0 (0%)	0.499
Grade II	2 (9.1%)	4 (19.0%)	3 (15.8%)	
Grade III	3 (13.6%)	3 (14.3%)	6 (31.6%)	
Grade IV	13 (59.1%)	13 (61.9%)	8 (42.1%)	
Unknown	4 (18.2%)	1 (4.8%)	2 (10.5%)	
**Treatment**				0.992
Previous operation	15 (68.2%)	10 (47.6%)	11 (57.9%)	
No previous operation	7 (31.8%)	11 (52.4%)	8 (42.1%)	
**Current treatment**				0.565
Oral contraceptives	10 (45.5%)	10 (47.6%)	7 (36.9%)	
GnRH agonist (Lucrin)	2 (9.1%)	2 (9.5%)	2 (10.5%)	
IUD	1 (4.5%)	0 (0%)	0 (0%)	
Oral contraceptives with IUD	2 (9.1%)	1 (4.8%)	2 (10.5%)	
Pain medication	0 (0%)	1 (4.8%)	2 (10.5%)	
Pain medication in addition to hormonal therapy	1 (4.5%)	2 (9.5%)	0 (0%)	
Different treatment	0 (0%)	2 (9.5%)	0 (0%)	
No treatment	6 (27.3%)	3 (14.3%)	6 (31.6%)	

1 FODMAP: fermentable oligo-, di-, mono-saccharides, and polyols.

2 Data not normally distributed, therefore shown as median with interquartile range (IQR).

3 Fibroids, mastopathy, adhesions because of previous operations.

4 Gastro-esophageal reflux, irritable bowel syndrome (IBS), syndrome of Gilbert, chronic urinary tract infection, and enlarged bladder.

5 Diabetes.

6 Migraine, hernia.

7 Hypothyroidism, Hashimoto.

8 Hypertension, immune thrombocytopenia (ITP), alpha thalassemia, and previous brain aneurysm.

9 Asthma/asthmatic bronchitis, eczema, and hay fever.

10 Depression, bipolar disease, attention deficit disorder (ADD), attention deficit hyperactivity disorder (ADHD), and chronic fatigue.

11 ASRM, American Society for Reproductive Medicine.

All participants adhering to a diet reported significantly less deep dyspareunia, dysuria, bloating, and tiredness after adhering to the diet for 6 months compared to their baseline scores (range *P* < 0.001 to *P* = 0.012). Participants adhering to the Low FODMAP diet reported significantly less dysuria (*P* = 0.015) and bloating (*P* < 0.001), whereas participants adhering to the endometriosis diet reported significant less bloating (*P* < 0.001) and tiredness (*P* = 0.002) after 6 months compared to their baseline scores. Participants in the control group reported no significantly different pain scores in endometriosis-related symptoms at 6 months follow-up (Supplementary Table S1). In addition, the influence of self-reported strictness score on pain was studied. No significant influence of dietary strictness on the reported pain scores was found (Supplementary Table S2).

When compared to the control group, analyzed longitudinally over the course of the 6 months follow-up period, participants adhering to a dietary intervention experienced significantly less bloating, with a mean difference (MD) of −0.84 (*P* = 0.049). When analyzing data according to the intention-to-treat principle, this difference lost statistical significance. In addition, compared to the control group, participants adhering to the Low FODMAP diet reported less deep dyspareunia (MD −1.15; *P* = 0.032) whereas participants adhering to the endometriosis diet reported less bloating (MD −0.99, *P* = 0.041). When analyzing the data according to the intention-to-treat principle, this MD in deep dyspareunia for women adhering to the Low FODMAP diet and bloating for women adhering to the endometriosis diet was smaller but nevertheless remained significant (Table 3).

**Table 3.** Difference in reported VAS scores for endometriosis-related symptoms between the dietary interventions and control group, compared longitudinally over the follow-up period of 6 months.

	Intervention (FODMAP^1^ or endometriosis diet) n = 43			Control n = 19			
Symptoms associated with endometriosis	Baseline VAS^2^ score (median, IQR)	Three-month VAS score (median, IQR)	Six-month VAS score (median, IQR)	Baseline VAS score (median, IQR)	Three-month VAS score (median, IQR)	Six-month VAS score (median, IQR)	Mean difference^3^ (95% CI) *P*-value
**Dysmenorrhea**	5.0 (8.0)	3.28 (3.53)	3.0 (6.0)	4.0 (7.0)	5.0 (7.0)	4.0 (7.0)	−0.36 (−1.45 to 0.73) **0.515**
**Deep dyspareunia**	4.0 (5.0)	2.41 (2.48)	1.0 (4.0)	3.5 (6.0)	4.0 (5.0)	1.0 (6.0)	−0.83 (−1.74 to 0.08) **0.075**
**Chronic Pelvic Pain**	2.0 (6.0)	2.34 (2.54)	3.0 (6.0)	2.5 (6.0)	1.0 (5.0)	2.0 (4.0)	−0.014 (−0.94 to 0.91) **0.976**
**Dysuria**	1.0 (4.0)	0.85 (1.81)	0.0 (1.0)	0.0 (3.0)	1.0 (1.0)	0.0 (1.0)	0.05 (−0.58 to 0.67) **0.880**
**Bloating**	8.0 (3.5)	3.39 (2.70)	3.0 (4.0)	6.0 (4.0)	6.0 (4.0)	5.0 (5.0)	−0.84 (−1.68 to −0.004) **0.049**
**Tiredness**	7.0 (4.0)	4.79 (2.58)	5.0 (3.0)	7.0 (2.8)	6.0 (5.0)	6.0 (6.0)	−0.04 (−0.88 to 0.80) **0.924**

	Low-FODMAP diet				Endometriosis diet			
	n = 22				n = 21			
Symptoms associated with endometriosis	Baseline VAS score (median, IQR)	Three-month VAS score (median, IQR)	Six-month VAS score (median, IQR)	Mean difference^4^ (95% CI) *P*-value	Baseline VAS score (median, IQR)	Three-month VAS score (median, IQR)	Six-month VAS score (median, IQR)	Mean difference^4^ (95% CI) *P*-value
**Dysmenorrhea**	4.0 (7.0)	1.0 (7.0)	2.0 (4.0)	−1.08 (−2.31 to 0.16) **0.087**	6.0 (7.0)	5.0 (8.0)	3.5 (7.0)	0.32 (−0.90 to 1.54) **0.606**
**Deep dyspareunia**	4.5 (6.0)	1.5 (3.0)	0.0 (4.0)	−1.15 (−2.2 to −0.10) **0.032**	4.0 (6.0)	2.0 (6.0)	2.0 (5.0)	−0.55 (−1.58 to 0.49) **0.298**
**Chronic pelvic pain**	2.0 (6.0)	1.0 (4.0)	4.0 (6.0)	−0.11 (−1.17 to 0.96) **0.845**	3.0 (6.0)	2.0 (5.0)	2.5 (5.0)	0.07 (−0.98 to 1.12) **0.894**
**Dysuria**	1.0 (3.0)	0.0 (0.0)	0.0 (0.0)	−0.19 (−0.90 to 0.53) **0.607**	1.0 (4.5)	0.0 (3.0)	0.0 (1.0)	0.26 (−0.44 to 0.97) **0.464**
**Bloating**	8.0 (3.8)	3.0 (4.25)	3.0 (4.5)	−0.69 (−1.66 to 0.27) **0.159**	7.0 (3.5)	2.0 (4.0)	2.0 (3.0)	−0.99 (−1.94 to −0.039) **0.041**
**Tiredness**	7.0 (4.0)	5.0 (5.0)	5.0 (5.0)	0.07 (−0.90 to 1.04) **0.884**	7.0 (5.0)	5.0 (4.0)	3.5 (2.8)	−0.15 (−1.10 to 0.81) **0.761**

Mean differences with interquartile ranges (IQR) were calculated using mixed models.

Bold values indicate *P*-values.

1 FODMAP: fermentable oligo-, di-, mono-saccharides, and polyols.

2 VAS: visual analogue scale (scale 0–10 cm).

3 Difference calculated between baseline, 12- and 24-week follow-up, between the two dietary interventions and control group.

4 Difference calculated between baseline, 12- and 24-week follow-up. Difference calculated between the Low FODMAP diet and control group, and between endometriosis diet and control group.

QoL was assessed using the EHP-30 questionnaire, and scores were calculated for all QoL domains. All participants adhering to a diet scored significantly better in the domains pain, powerlessness, emotional wellbeing, self-image, work life, and sexual intercourse after adhering to the diet for 6 months compared to their baseline QoL scores (range *P* < 0.001 to *P* = 0.023). Participants adhering to the Low FODMAP diet reported significantly better scores after 6 months compared to their baseline scores in the QoL domains pain, powerlessness, and work life (*P* = 0.007, *P* = 0.002, and *P* = 0.013, respectively) whereas participants adhering to the endometriosis diet reported significantly better scores in the QoL domains powerlessness, emotional wellbeing, self-image, and treatment (range *P* < 0.001 to *P* = 0.023). Participants in the control group reported no significant differences in QoL after 6 months (Supplementary Table S3).

When comparing them to the control group, analyzed longitudinally over the course of the 6 months follow-up period, participants adhering to a dietary intervention reported significantly better scores in the QoL domains social support (MD −11.26; *P* = 0.004) and medical profession (MD −17.96; *P* = 0.005). When analyzing data according to the intention-to-treat principle, only a significant difference in social support remained. Compared to the control group, participants adhering to the Low FODMAP diet reported a better score in the QoL domain medical profession (MD −17.14, *P* = 0.018) whereas participants adhering to the endometriosis diet reported significant scores in the QoL domains social support (MD −15.47; *P* < 0.001) and medical profession (MD −18.71; *P* = 0.010). When analyzing the data according to the intention-to-treat principle, these MDs in medical profession for women adhering to the Low FODMAP diet and in social support and medical profession for women adhering to the endometriosis diet were smaller but nevertheless remained significant (Table 4).

**Table 4.** Difference in quality of life domains between the dietary interventions and control group, compared longitudinally over the follow-up period of 6 months.

	Intervention (FODMAP^1^ or endometriosis diet)			Control			
	n = 43			n = 19			
Domains of quality of life measured using the EHP-30^2^	Baseline EHP-30 score (median, IQR)	Three-month EHP-30 score (median, IQR)	Six-month EHP-30 score (median, IQR)	Baseline EHP-30 score (median, IQR)	Three-month EHP-30 score (median, IQR)	Six-month EHP-30 score (median, IQR)	Mean difference^3^ (95% CI) *P*-value
**Pain**	42.0 (29.0)	25.0 (39.2)	22.7 (37.5)	28.4 (33.5)	31.8 (13.6)	22.7 (25.0)	−2.41 (−8.97 to 4.15) **0.469**
**Powerlessness**	50.0 (30.2)	20.8 (29.2)	16.7 (29.2)	35.4 (31.3)	37.5 (12.5)	29.2 (25.0)	−4.31 (−10.86 to 2.24) **0.195**
**Emotional wellbeing**	33.3 (33.3)	25.0 (32.3)	20.8 (32.3)	31.3 (26.0)	37.5 (25.0)	16.7 (25.0)	−1.83 (−7.53 to 3.88) **0.528**
**Social support**	34.4 (39.1)	37.5 (43.8)	28.1 (31.3)	43.8 (39.1)	50.0 (31.3)	37.5 (18.8)	−11.26 (−18.77 to −3.74) **0.004**
**Self-image**	50.0 (43.8)	25.0 (41.7)	25.0 (39.6)	41.7 (27.1)	33.3 (33.3)	25.0 (58.3)	−1.45 (−9.59 to −6.69) **0.726**
**Work life**	25.00 (40.0)	7.50 (28.75)	5.0 (25.0)	10.00 (15.0)	15.0 (18.75)	10.0 (22.5)	1.89 (−4.77 to 8.55) **0.576**
**Children**	0.0 (37.5)	0.0 (21.9)	0.0 (9.4)	0.0 (0.0)	0.0 (0.0)	0.0 (0.0)	3.66 (−3.22 to 10.53) **0.295**
**Sexual intercourse**	45.0 (51.3)	25.0 (40.0)	27.5 (47.5)	50.0 (42.5)	50.0 (45.0)	15.0 (75.0)	−6.97 (−15.79 to 1.85) **0.120**
**Medical profession**	6.3 (37.5)	15.6 (31.3)	12.5 (37.5)	18.8 (37.5)	25.0 (56.3)	40.6 (39.1)	−17.96 (−30.26 to −5.66) **0.005**
**Treatment**	41.7 (39.6)	37.5 (33.3)	33.3 (41.7)	33.3 (25.0)	25.0 (45.8)	25.0 (66.7)	7.17 (−2.21 to 16.55) **0.132**
**Infertility**	71.9 (32.8)	87.5 (–)	56.3 (56.3)	87.5 (31.3)	75.0 (25.0)	81.3 (–)	−9.37 (−25.60 to 6.86) **0.243**

	Low-FODMAP diet				Endometriosis diet			
	n = 22				n = 21			
Domains of quality of life measured using the EHP-30	Baseline EHP-30 score (median, IQR)	Three-month EHP-30 score (median, IQR)	Six-month EHP-30 score (median, IQR)	Mean difference^4^ (95% CI) *P*-value	Baseline EHP-30 score (median, IQR)	Three-month EHP-30 score (median, IQR)	Six-month EHP-30 score (median, IQR)	Mean difference^4^ (95% CI) *P*-value
**Pain**	43.2 (28.4)	20.5 (34.1)	15.9 (31.8)	−3.60 (−11.15 to 3.95) **0.348**	40.9 (37.5)	29.5 (39.8)	22.7 (50.0)	−1.36 (−8.80 to 6.09) **0.720**
**Powerlessness**	54.2 (27.1)	20.8 (41.7)	16.7 (37.5)	−1.38 (−8.90 to 6.14) **0.718**	45.8 (43.8)	20.8 (27.1)	16.7 (35.4)	−6.80 (−14.20 to 0.61) **0.072**
**Emotional wellbeing**	37.5 (37.5)	25.0 (37.5)	20.8 (37.5)	0.81 (−5.72 to 7.34) **0.807**	33.3 (33.3)	25.0 (20.8)	20.8 (27.1)	−4.15 (−10.58 to 2.28) **0.204**
**Social support**	31.3 (40.6)	43.8 (43.8)	31.3 (50.0)	−6.93 (−15.48 to 1.62) **0.111**	37.5 (40.6)	12.5 (46.9)	25.0 (34.4)	−15.47 (−23.89 to −7.06) **<0.001**
**Self-image**	50.0 (50.0)	25.0 (41.7)	33.3 (58.3)	1.08 (−8.26 to 10.42) **0.820**	50.0 (37.5)	33.3 (37.5)	16.7 (29.2)	−3.98 (−13.18 to 5.23) **0.395**
**Work life**	30.0 (40.0)	5.0 (20.0)	5.0 (25.0)	3.12 (−4.59 to 10.83) **0.425**	22.5 (38.8)	10.0 (32.5)	5.0 (10.0)	0.86 (−6.77 to 8.49) **0.824**
**Children**	0.0 (43.8)	0.0 (12.5)	0.0 (0.0)	4.40 (−3.52 to 12.32) **0.274**	0.0 (37.5)	0.0 (25.0)	0.0 (18.8)	2.99 (−4.80 to 10.78) **0.450**
**Sexual intercourse**	45.0 (50.0)	20.0 (40.0)	30.0 (50.0)	−9.97 (−20.09 to 0.14) **0.053**	45.0 (47.5)	30.0 (35.0)	20.0 (50.0)	−4.31 (−14.27 to 5.66) **0.394**
**Medical profession**	6.3 (37.5)	15.6 (53.1)	12.5 (18.8)	−17.14 (−31.27 to −3.00) **0.018**	0.0 (43.8)	12.5 (31.3)	6.3 (42.2)	−18.71 (−32.79 to −4.63) **0.010**
**Treatment**	25.0 (37.5)	33.3 (33.3)	41.7 (37.5)	5.35 (−5.12 to 15.82) **0.312**	50.0 (25.0)	41.7 (41.7)	33.3 (25.0)	9.33 (−1.35 to 20.01) **0.086**
**Infertility**	68.8 (–)	68.8 (–)	56.3 (–)	−11.88 (−31.66 to 7.90) **0.224**	78.1 (–)	x	56.3 (56.3)	−6.08 (−26.87 to 14.70) **0.550**

Mean differences with interquartile ranges (IQRs) were calculated using mixed models.

Bold values indicate *P*-values.

1 FODMAP: fermentable oligo-, di-, mono-saccharides, and polyols.

2 EHP-30 Questionnaire: endometriosis health profile 30 questionnaire (range 0–100).

3 Difference calculated between baseline, 12- and 24-week follow-up, between the two dietary interventions and control group.

4 Difference calculated between baseline, 12- and 24-week follow-up. Difference calculated between the Low FODMAP diet and control group, and between endometriosis diet and control group.

Participants adhering to a diet experienced better gastro-intestinal health, measured using the GIQLI questionnaire, after adhering to the diet for 6 months compared to their baseline scores (*P* < 0.001) (Supplementary Table S4). However, when comparing data to the control group, analyzed longitudinally over the course of the 6 months follow-up period, participants adhering to a dietary intervention reported no improvement in gastro-intestinal health (Table 5).

**Table 5.** Difference in gastro-intestinal quality of life, measured using the gastro-intestinal quality of life index, between the dietary interventions and control group, compared longitudinally over the follow-up period of 6 months.

GIQLI score^2^	Intervention (FODMAP^1^ or endometriosis diet)			Control			
	n = 43			n = 19			
	Baseline GIQLI score (mean, SD)	Three-month GIQLI score (mean, SD)	Six-month GIQLI score (mean, SD)	Baseline GIQLI score (mean, SD)	Three-month GIQLI score (mean, SD)	Six-month GIQLI score (mean, SD)	Mean difference^3^ (95% CI) *P*-value
	127.35 (14.67)	136.85 (12.79)	139.03 (11.09)	134.29 (14.79)	134.11 (9.34)	133.27 (13.82)	1.31 (−5.49 to 2.88) **0.539**

GIQLI score	Low-FODMAP diet				Endometriosis diet			
	n = 22				n = 21			
	Baseline GIQLI score (mean, SD)	Three-month GIQLI score (mean, SD)	Six-month GIQLI score (mean, SD)	Mean difference^4^ (95% CI) *P*-value	Baseline GIQLI score (mean, SD)	Three-month GIQLI score (mean, SD)	Six-month GIQLI score (mean, SD)	Mean difference^4^ (95% CI) *P*-value
	122.68 (14.36)	135.11 (13.89)	136.74 (9.81)	1.58 (−6.32 to 3.16) **0.511**	132.24 (13.66)	138.50 (11.77)	141.10 (11.86)	3.88 (−0.82 to 8.57) **0.105**

Mean differences with interquartile ranges (IQR) were calculated using mixed models.

Bold values indicate *P*-values.

1 FODMAP: fermentable oligo-, di-, mono-saccharides, and polyols.

2 GIQLI: Gastro-Intestinal Quality of Life Index (range 0–144).

3 Difference calculated between baseline, 12- and 24-week follow-up, between the two dietary interventions and control group.

4 Difference calculated between baseline, 12- and 24-week follow-up. Difference calculated between the Low FODMAP diet and control group, and between endometriosis diet and control group.

### Results for patient experiences

Participants had different motivations to participate in the dietary intervention. Mostly, they hoped it would decrease their pain symptoms, would reduce their bowel symptoms, such as flatulence and bloating, and would optimize their general health. At the start of their diet, most participants found adherence to the diet difficult whereas by the 6 months follow-up they found adherence to the diet less difficult (difficulty ‘normal’), indicating normalization toward dealing with a diet in daily life (Low FODMAP diet *P* < 0.001 and endometriosis diet *P* = 0.017). Difficulties most frequently reported were high costs, the time-consuming element and other difficulties such as adherence to the diet in a social setting, i.e. when going out for dinner or eating with friends and family. Almost all participants completed 6 months adherence to their diet (40/43). Three participants decided to discontinue their diet prematurely because of personal circumstances such as moving to another country (n = 1) or an illness in the family (n = 1). One participant did not give any details regarding her personal circumstances leading the discontinuation of her diet. There was no cross-over by participants to the other dietary intervention. No participants experienced side effects from their chosen dietary intervention. Finally, the majority of participants expressed that they would (partially) continue their diet at the end of the study (80.6% in endometriosis diet versus 81.8% Low FODMAP diet) (Supplementary Table S5).

## Discussion

This prospective single-center pilot study aimed to investigate the effect of two dietary interventions on endometriosis-related symptoms and QoL, and compare it to a control group, analyzed longitudinally over the course of a 6-month follow-up period. Our results suggest that women adhering to a dietary intervention experience less non-cyclical deep dyspareunia, cyclical dysuria, bloating, and tiredness after 6 months*.* Other (non-)cyclical symptoms associated with endometriosis, such as dysmenorrhea and chronic pelvic pain, were not significantly improved. When comparing participants adhering to a dietary intervention to participants in the control group, analyzed longitudinally over the course of the 6-month follow-up period, only a significant difference in bloating remained. Our results additionally suggest that women adhering to a dietary intervention reported better EHP-30 scores in the QoL domains pain, powerlessness, emotional wellbeing, self-image, work life, and sexual intercourse after 6 months*.* When comparing participants adhering to a dietary intervention to participants in the control group, only a significant difference in social support and medical profession remained. There was a high level of satisfaction with the dietary guidance. Ultimately, 35 out of 43 participants wanted to continue their diet, at least partially, at the end of the 6-month follow-up period.

### Clinical implications

To our knowledge, this pilot study is the first to examine the effect of both the Low FODMAP diet and endometriosis diet on endometriosis-related symptoms and QoL in women with endometriosis. In a previous retrospective study using a nationwide survey, our study group found that Dutch women adhering to the endometriosis diet reported significantly improved QoL, especially when there was strict adherence to the diet (van Haaps *et al.*, 2023). Although a positive impact of self-reported diet strictness on pain scores was not observed in our current study, potentially owing to the small sample size, we did observe a positive association between dietary adherence and both pain scores and QoL.

Our study suggests that adherence to a dietary intervention could reduce both (non-)cyclical endometriosis-related symptoms and gastro-intestinal symptoms such as bloating. Previously, an overlap was seen between symptoms associated with endometriosis and symptoms associated with IBS (Moore *et al.*, 2017). Jess *et al.* observed that endometriosis was more frequently associated with gastro-intestinal dysfunction, as found in IBS. In their nationwide Danish cohort of 37 661 women, a significant association between endometriosis and IBS (standardized incidence ratio 1.5 (95% CI 1.3–1.7)) was observed (Jess *et al.*, 2012). Additionally, in an Australian cross-sectional survey (N = 484) on self-management strategies among women surgically diagnosed with endometriosis, 44% of respondents adhered to a dietary intervention such as the Low FODMAP diet, gluten free- or lactose-free diet. They found that these dietary interventions were effective in reducing endometriosis-related pelvic pain, as well as gastro-intestinal symptoms such as abdominal discomfort (i.e. flatulence, bloating, and diarrhea) (Armour *et al.*, 2021). Comparable results to Armour *et al.* (2021) were found in a study evaluating the effect of the Low FODMAP diet on endometriosis-related and gastro-intestinal symptoms, in women with endometriosis and IBS (Moore *et al.*, 2017). Finally, Sesti *et al.* (2007) recently studied the effect of treatment on pain symptoms and QoL after conservative pelvic surgery for endometriosis grade III–IV. They found that post-surgical treatment with a dietary intervention was similarly effective as post-surgical treatment with a GnRH-agonist or continuous oral contraceptives (Sesti *et al.*, 2007). These studies, in addition to our findings, support the hypothesis that a dietary intervention could improve both endometriosis-related symptoms and gastro-intestinal symptoms. Therefore, women diagnosed both with endometriosis and a gastro-intestinal diagnosis might benefit most from a dietary intervention. However, since no additional analyses on this were performed in our study, we cannot state this implication with certainty.

Contrary to our findings, a cross-sectional survey study on the effect of different, frequently applied diets in Dutch patients with endometriosis did not show any significant differences in QoL between women adhering to a diet and women not adhering to a diet. However, they did find that 224 of the 314 specific dietary adjustments (71.3%) reported by the participants were considered to contribute to the reduction of the participants’ chronic endometriosis symptoms (Krabbenborg *et al.*, 2021).

Our pilot study additionally found that adherence to a dietary intervention led to an improvement in QoL*.* According to Soliman *et al.* (2017), experiencing more pelvic pain or menstrual cramping, anxiety or stress, fatigue, bloating, and intermenstrual bleeding was associated with a lower QoL (Soliman *et al.*, 2017). This might explain why our participants, with improved pain scores, additionally reported improved QoL scores. Furthermore, women participating in our study received extensive guidance by the dietician in training for the first 3 months and were provided with tools to optimally adhere to their chosen diet. The active role that participants took in managing their endometriosis-related symptoms may have contributed to a greater sense of control over their endometriosis, possibly contributing to an improved QoL. Previous research has shown that a person-centered approach, in which women were able to take an active role in their disease management and worked together with the healthcare provider aiming to improve their symptoms, resulted in a greater feeling of control over their symptoms and more positive healthcare provider experiences (O'Hara *et al.*, 2019). These studies, in addition to our findings, show that a dietary intervention can improve QoL in women with endometriosis, both through reducing pain symptoms and by empowering women to take an active role in their disease management.

While previous studies have described the positive effects of dietary interventions, such as the Low FODMAP diet, on pain and QoL, they might also have negative effects. There is evidence that the Low FODMAP diet may induce profound changes in the composition and functioning of the gut microbiota. However, there is still a lot of uncertainty regarding this effect. In addition, evidence regarding the long-term effects of the Low FODMAP diet is limited, since most previous studies only had a follow-up period of <12 weeks (Staudacher and Whelan, 2017). Regarding the endometriosis diet, its impact on endometriosis-related symptoms, the long-term effects, and possible negative effects on health have not been investigated in previous studies. Therefore, the endometriosis diet is currently not advised by dieticians in the Netherlands (Huijs and Nap, 2020). In the present study, we handled a follow-up period of 6 months (24 weeks) for both diets. However, since this was a pilot study lacking a calculation of sample size, we cannot attribute with complete certainty all effects on pain and QoL to the dietary interventions. To determine long-term effects with more certainty in future studies, it could be recommended to extend the follow-up period to at least 6 months, similar to our pilot study. This underlines the importance of additional research on the efficacy and safety, including possible negative effects on health, of dietary interventions on endometriosis-related symptoms and associated QoL, with a longer follow-up period.

Adherence to the endometriosis diet or Low FODMAP diet can be associated with higher costs. According to a British study, adherence to the Low FODMAP diet for a period of 6 weeks cost an additional €175.07 (£139.20; exchange rate £1 = €1.2577) (Whigham *et al.*, 2015). Although the costs of the endometriosis diet have never been studied, the diet is very similar to the Mediterranean diet, which costs an additional €0.71 per 1000 kcal, compared to a standard diet (Saulle *et al.*, 2013). In our study, dietary guidance was provided free of charge for participants. However, dietary guidance normally costs between €63 and €73 per hour for both dietary interventions in our study. It is thought that an average of two to four guidance sessions, with a total of 5 hours, are needed to optimally adhere to a diet. These costs are not refunded by health insurance in most countries. In the Netherlands only 3 h of dietary guidance are covered by basic health insurance. However, additional costs for adherence to a diet on medical indication, such as higher grocery costs, are tax-deductible in the Netherlands, which can reduce the dietary costs significantly. In 2022, annual dietary costs were tax deductible up to €1350 for the endometriosis diet and up to €1050 for the Low FODMAP diet.

### Strengths and limitations

Our study has several strengths. First, very few women discontinued adherence to their chosen diet prematurely and withdrew from study participation. Both the extensive guidance and the information materials contributed to this positive result. The fact that the significant differences were smaller or no longer seen when data were analyzed according to the intention-to-treat principle, where women adhering to their dietary intervention for only 3–6 weeks were included, underlines the importance of adherence to a dietary intervention for a longer period of time. In addition, a guideline for adherence to the endometriosis diet was developed and the composition of the diet was standardized. Finally, our follow-up period spanned a longer time period than previous studies. Our follow-up period entailed 24 weeks, whereas other studies had a follow-up period of 12 weeks at most. This contributes to increasing knowledge on the long-term effects of the dietary adjustments when compared to no diet.

Our study also has several limitations. Since this was a pilot study designed to explore the feasibility of treating women with endometriosis with one of two dietary interventions and data were lacking for effect sizes, we were not able to calculate a sample size. Moreover, this was a patient preference study where randomization was not applied. Although this might have induced selection bias, we valued a strong motivation to adhere to a diet, accepting the risk that the participant pool may not represent the target population. We were not able to generalize our conclusions, as would be the case if the trial was conducted as a randomized controlled trial (RCT). However, we believe that an RCT in dietary interventions will not be feasible as dietary interventions require a very high motivation from participants. Furthermore, the current study design is similar to clinical practice where women decide themselves whether they want to adhere to a dietary intervention or not.

We cannot exclude that the Hawthorne effect somewhat influenced our results. Since women were aware they participated in a trial, adjusted their behavior by altering their diet, and received attention from the dietician in training for 6 months, this could have favorably influenced their reported pain scores and QoL (Sedgwick and Greenwood, 2015). The effect of the added attention from a dietician would be common practice for all dietary interventions and is impossible to separate from the effect of the diet itself. However, since we advise always having support from a dietician when adjusting a diet, it is not necessary to divide the effects. Finally, some women found it hard to quantify their pain retrospectively using pain scores. They still experienced pain, resulting in them scoring their pain quite high. However, their pain occurred much less frequently, possibly making the quantification of pain using recalled VAS-scores difficult. Van Barneveld *et al.* (2023) have recently developed an electronic instrument to objectify symptoms in women with endometriosis. In their study, women were asked to register their pain scores at random moments over a period of 1 week, 10 times a day. They found that these random pain scores were significantly lower compared to the reported ‘recalled’ pain scores requested at the end of the week. Perhaps this pain tool would have been more effective in objectifying the pain scores of our participants (van Barneveld *et al.*, 2023). Despite these limitations, our study paints a promising picture of the effect of the Low FODMAP diet and endometriosis diet on endometriosis-related symptoms and QoL.

## Conclusion

With our study, we have aimed to provide a more scientifically substantiated answer to the question in daily practice of women diagnosed with endometriosis, of whether they should apply a dietary intervention for their symptoms. Our study suggests that women adhering to a diet experience less pain and improved QoL after 6 months. When comparing it to the control group, analyzed longitudinally over the 6 months follow-up period, only significantly less bloating and improved scores in the QoL domains medical treatment and social support were seen in women adhering to a dietary intervention. Therefore, applying a dietary intervention to women diagnosed with endometriosis could be discussed during counseling, especially when (hormonal) therapy and surgery fail to completely eliminate their symptoms and there is a wish for self-management of their chronic pain complaints. However, several possibly problematic issues should be discussed with the patient such as the additional costs and the uncertainty regarding possible negative effects. Therefore, women should be encouraged to seek help from a dietician when applying a new diet, also to ensure a fully fledged diet. Since this is a pilot study without a calculated sample size, we cannot attribute the positive effects with complete certainty to a dietary intervention. For future studies calculating a sample size, based on the findings of this study a follow-up period of at least 6 months, and increasing knowledge regarding possible negative effects of the dietary interventions, is recommended.

## Supplementary Material

dead214_Supplementary_Table_S1

dead214_Supplementary_Table_S2

dead214_Supplementary_Table_S3

dead214_Supplementary_Table_S4

dead214_Supplementary_Table_S5

## Data Availability

The data underlying this article will be shared on reasonable request to the corresponding author.
