# Supplementary material for: The effect of dietary interventions on pain and quality of life in women diagnosed with endometriosis: a prospective study with control group
Source: Hum Reprod. 2023 Oct 24;38(12):2433–46. doi: 10.1093/humrep/dead214 (PMC10754387; doi:10.1093/humrep/dead214)
Supplement: dead214_Supplementary_Table_S1 [file dead214_supplementary_table_s1.pdf]

OXFORD

human reproduction

Human Reproduction, 2023, 1–1
https://doi.org/10.1093/humrep/dead214
Supplementary Data

Supplementary Table S1. Difference in reported VAS scores for endometriosis-related symptoms reported by women adhering to the dietary interventions and by women part of the control group.

	Intervention (Low FODMAP[1] or endometriosis diet)			Control		
	N = 43			N = 19		
**Symptoms associated with endometriosis**	**Baseline VAS[2] score (median, IQR[3])**	**Six-month VAS score (median, IQR)**	***P*-value[4]**	**Baseline VAS score (median, IQR)**	**Six-month VAS score (median, IQR)**	***P*-value[4]**
**Dysmenorrhea**	5.0 (8.0)	3.0 (6.0)	0.083	4.0 (7.0)	4.0 (7.0)	0.634
**Deep dyspareunia**	4.0 (5.0)	1.0 (4.0)	0.012	3.5 (6.0)	1.0 (6.0)	0.424
**Chronic pelvic pain**	2.0 (6.0)	3.0 (6.0)	0.707	2.5 (6.0)	2.0 (4.0)	0.985
**Dysuria**	1.0 (4.0)	0.0 (1.0)	0.005	0.0 (3.0)	0.0 (1.0)	0.256
**Bloating**	8.0 (3.5)	3.0 (4.0)	<0.001	6.0 (4.0)	5.0 (5.0)	0.487
**Tiredness**	7.0 (4.0)	5.0 (3.0)	<0.001	7.0 (2.8)	6.0 (6.0)	0.200

	Low FODMAP diet			Endometriosis diet		
	N = 22			N = 21		
**Symptoms associated with endometriosis**	**Baseline VAS score (median, IQR)**	**Six-month VAS score (median, IQR)**	***P*-value[4]**	**Baseline VAS score (median, IQR)**	**Six-month VAS score (median, IQR)**	***P*-value[4]**
**Dysmenorrhea**	4.0 (7.0)	2.0 (4.0)	0.111	6.0 (7.0)	3.5 (7.0)	0.298
**Deep dyspareunia**	4.5 (6.0)	0.0 (4.0)	0.066	4.0 (6.0)	2.0 (5.0)	0.078
**Chronic pelvic pain**	2.0 (6.0)	4.0 (6.0)	0.433	3.0 (6.0)	2.5 (5.0)	0.809
**Dysuria**	1.0 (3.0)	0.0 (0.0)	0.015	1.0 (4.5)	0.0 (1.0)	0.105
**Bloating**	8.0 (3.8)	3.0 (4.5)	<0.001	7.0 (3.5)	2.0 (3.0)	<0.001
**Tiredness**	7.0 (4.0)	5.0 (5.0)	0.087	7.0 (5.0)	3.5 (2.8)	0.002

Differences were calculated between baseline and 6-month follow-up.
[1] FODMAP: fermentable oligo-, di-, mono-saccharides, and polyols.
[2] VAS: visual analogue score (scale 0–10 cm).
[3] IQR: interquartile range.
[4] Calculated using the Mann–Whitney *U* test because of non-normal distributed data.
