# Supplementary material for: The effect of dietary interventions on pain and quality of life in women diagnosed with endometriosis: a prospective study with control group
Source: Hum Reprod. 2023 Oct 24;38(12):2433–46. doi: 10.1093/humrep/dead214 (PMC10754387; doi:10.1093/humrep/dead214)
Supplement: dead214_Supplementary_Table_S2 [file dead214_supplementary_table_s2.pdf]

OXFORD
human reproduction

*Human Reproduction*, 2023, 1–1
https://doi.org/10.1093/humrep/dead214
Supplementary Data

**Supplementary Table S2.** The influence of self-reported strictness scores on endometriosis-related pain, expressed using VAS scores.

| | Low-FODMAP[1] diet (N = 22) | | | | Endometriosis diet (N = 21) | | | |
|---|---|---|---|---|---|---|---|---|
| **Self-reported strictness score (mean, SD)** | **Three-month follow-up** | | **Six-month follow-up** | | **Three-month follow-up** | | **Six-month follow-up** | |
| | 8.75 (0.91) | | 7.47 (1.55) | | 7.67 (1.78) | | 7.23 (0.83) | |
| **Symptoms associated with endometriosis** | **Three-month follow-up** | | **Six-month follow-up** | | **Three-month follow-up** | | **Six-month follow-up** | |
| | **Effect of strictness on pain scores (95% CI)** | **P-value** | **Effect of strictness on pain scores (95% CI)** | **P-value** | **Effect of strictness on pain scores (95% CI)** | **P-value** | **Effect of strictness on pain scores (95% CI)** | **P-value** |
| **Dysmenorrhea** | −0.76 (−2.71 to 1.19) | **0.423** | 0.13 (−0.57 to 0.83) | **0.697** | 0.13 (−0.96 to 1.21) | **0.810** | 0.11 (−0.72 to 0.94) | **0.791** |
| **Dyspareunia** | −0.83 (−2.08 to 0.42) | **0.177** | 0.17 (−0.68 to 1.02) | **0.676** | −0.11 (−0.94 to 0.72) | **0.781** | 0.36 (−0.29 to 1.01) | **0.259** |
| **Chronic Pelvic Pain** | −0.80 (−2.40 to 0.79) | **0.304** | 0.40 (−0.44 to 1.23) | **0.329** | 0.32 (−0.44 to 1.07) | **0.387** | 0.54 (−0.12 to 1.20) | **0.103** |
| **Dysurea** | −0.15 (−1.32 to 1.02) | **0.789** | 0.19 (−0.23 to 0.61) | **0.353** | −0.29 (−0.81 to 0.24) | **0.266** | −0.001 (−0.51 to 0.51) | **0.996** |
| **Bloating** | −1.25 (−2.71 to 0.21) | **0.088** | 0.29 (−0.39 to 0.98) | **0.377** | 0.10 (−0.79 to 0.99) | **0.818** | −0.18 (−0.62 to 0.25) | **0.384** |
| **Tiredness** | −0.47 (−2.34 to 1.40) | **0.598** | −0.12 (−0.97 to 0.73) | **0.769** | 0.14 (−0.60 to 0.88) | **0.699** | 0.14 (−0.43 to 0.71) | **0.617** |

The effect, with 95% confidence intervals (95% CI) were calculated using a logistic regression.
Bold values indicate *P*-values.
[1] FODMAP: fermentable oligo-, di-, mono-saccharides, and polyols.
