# Supplementary material for: The effect of dietary interventions on pain and quality of life in women diagnosed with endometriosis: a prospective study with control group
Source: Hum Reprod. 2023 Oct 24;38(12):2433–46. doi: 10.1093/humrep/dead214 (PMC10754387; doi:10.1093/humrep/dead214)
Supplement: dead214_Supplementary_Table_S3 [file dead214_supplementary_table_s3.pdf]

OXFORD

human reproduction

Human Reproduction, 2023, 1–1
https://doi.org/10.1093/humrep/dead214
Supplementary Data

Supplementary Table S3. Difference in QoL reported by women adhering to the dietary interventions and women part of the control group.

Domains of quality of life measured using the EHP-30[2]	Intervention (Low FODMAP[1] or endometriosis diet) N = 43 Baseline EHP-30 score (median, IQR[3])	Intervention N = 43 Six-month EHP-30 score (median, IQR)	Intervention P-value[4]	Control N = 19 Baseline EHP-30 score (median, IQR)	Control N = 19 Six-month EHP-30 score (median, IQR)	Control P-value[4]
Pain	42.0 (29.0)	22.7 (37.5)	0.005	28.4 (33.5)	22.7 (25.0)	0.302
Powerlessness	50.0 (30.2)	16.7 (29.2)	<0.001	35.4 (31.3)	29.2 (25.0)	0.261
Emotional wellbeing	33.3 (33.3)	20.8 (32.3)	0.003	31.3 (26.0)	16.7 (25.0)	0.156
Social support	34.4 (39.1)	28.1 (31.3)	0.150	43.8 (39.1)	37.5 (18.8)	0.884
Self-image	50.0 (43.8)	25.0 (39.6)	<0.001	41.7 (27.1)	25.0 (58.3)	0.637
Work life	25.00 (40.0)	5.0 (25.0)	0.002	10.00 (15.0)	10.0 (22.5)	0.790
Children	0.0 (37.5)	0.0 (9.4)	0.265	0.0 (0.0)	0.0 (0.0)	0.794
Sexual intercourse	45.0 (51.3)	27.5 (47.5)	0.023	50.0 (42.5)	15.0 (75.0)	0.156
Medical profession	6.3 (37.5)	12.5 (37.5)	0.581	18.8 (37.5)	40.6 (39.1)	0.254
Treatment	41.7 (39.6)	33.3 (41.7)	0.250	33.3 (25.0)	25.0 (66.7)	0.831
Infertility	71.9 (32.8)	56.3 (56.3)	0.255	87.5 (31.3)	81.3 (-)	0.879

Symptoms associated with endometriosis	Low FODMAP diet N = 22 Baseline EHP-30 score (median, IQR)	Low FODMAP diet N = 22 Six-month EHP-30 score (median, IQR)	Low FODMAP diet P-value[4]	Endometriosis diet N = 21 Baseline EHP-30 score (median, IQR)	Endometriosis diet N = 21 Six-month EHP-30 score (median, IQR)	Endometriosis diet P-value[4]
Pain	43.2 (28.4)	15.9 (31.8)	0.007	40.9 (37.5)	22.7 (50.0)	0.166
Powerlessness	54.2 (27.1)	16.7 (37.5)	0.002	45.8 (43.8)	16.7 (35.4)	0.014
Emotional wellbeing	37.5 (37.5)	20.8 (37.5)	0.073	33.3 (33.3)	20.8 (27.1)	0.022
Social support	31.3 (40.6)	31.3 (50.0)	0.723	37.5 (40.6)	25.0 (34.4)	0.126
Self-image	50.0 (50.0)	33.3 (58.3)	0.127	50.0 (37.5)	16.7 (29.2)	<0.001
Work life	30.0 (40.0)	5.0 (25.0)	0.013	22.5 (38.8)	5.0 (10.0)	0.056
Children	0.0 (43.8)	0.0 (0.0)	0.389	0.0 (37.5)	0.0 (18.8)	0.451
Sexual intercourse	45.0 (50.0)	30.0 (50.0)	0.153	45.0 (47.5)	20.0 (50.0)	0.079
Medical profession	6.3 (37.5)	12.5 (18.8)	0.423	0.0 (43.8)	6.3 (42.2)	0.830
Treatment	25.0 (37.5)	41.7 (37.5)	0.924	50.0 (25.0)	33.3 (25.0)	0.023
Infertility	68.8 (-)	56.3 (-)	0.767	78.1 (-)	56.3 (56.3)	0.355

Differences were calculated between baseline and 6-month follow-up.
[1] FODMAP: fermentable oligo-, di-, mono-saccharides, and polyols.
[2] EHP-30 questionnaire: Endometriosis Health Profile 30 questionnaire (range 0–100).
[3] IQR: Interquartile Range.
[4] Calculated using the Mann–Whitney *U* test because of non-normal distributed data.
