# Supplementary material for: The effect of dietary interventions on pain and quality of life in women diagnosed with endometriosis: a prospective study with control group
Source: Hum Reprod. 2023 Oct 24;38(12):2433–46. doi: 10.1093/humrep/dead214 (PMC10754387; doi:10.1093/humrep/dead214)
Supplement: dead214_Supplementary_Table_S4 [file dead214_supplementary_table_s4.pdf]

Human Reproduction, 2023, 1–1
https://doi.org/10.1093/humrep/dead214
Supplementary Data

Supplementary Table S4. Difference in gastro-intestinal quality of life, measured using the Gastro-Intestinal Quality of Life Index (GIQLI) reported by women adhering to the dietary interventions and women part of the control group.

	Intervention (Low FODMAP[1,*] or endometriosis diet) n = 43			Control n = 19		
GIQLI score[2,*]	Baseline GIQLI score (mean, SD)	Six-month GIQLI score (mean, SD)	*P*-value	Baseline GIQLI score (mean, SD)	Six-month GIQLI score (mean, SD)	*P*-value
	127.35 (14.67)	139.03 (11.09)	<0.001	134.29 (14.79)	133.27 (13.82)	0.570

Low FODMAP diet N = 22			Endometriosis diet N = 21		
Baseline GIQLI score (mean, SD)	Six-month GIQLI score (mean, SD)	*P*-value	Baseline GIQLI score (mean, SD)	Six-month GIQLI score (mean, SD)	*P*-value
122.68 (14.36)	136.74 (9.81)	0.001	132.24 (13.66)	141.10 (11.86)	0.029

Differences were calculated between baseline and 6-month follow-up. Differences were calculated using Mann–Whitney *U* test.
1 FODMAP: fermentable oligo-, di-, mono-saccharides, and polyols.
2 GIQLI: Gastro-Intestinal Quality of Life Index (range 0–144).
