# Supplementary material for: The effect of dietary interventions on pain and quality of life in women diagnosed with endometriosis: a prospective study with control group
Source: Hum Reprod. 2023 Oct 24;38(12):2433–46. doi: 10.1093/humrep/dead214 (PMC10754387; doi:10.1093/humrep/dead214)
Supplement: dead214_Supplementary_Table_S5 [file dead214_supplementary_table_s5.pdf]

OXFORD

human reproduction

*Human Reproduction*, 2023, 1–1

https://doi.org/10.1093/humrep/dead214

Supplementary Data

**Supplementary Table S5.** Experiences on the appliance of their chosen diet, reported by participants adhering to either the Low FODMAP diet or endometriosis diet.

| | Low FODMAP[1] diet | | | Endometriosis diet | | |
|---|---|---|---|---|---|---|
| **Descriptives** | | **(N = 22)** | | | **(N = 21)** | |
| **Previous dietary guidelines[2] (%)** | | 10 (45.5%) | | | 12 (57.1%) | |
| **Experience of the diet (%)** | **Three-week follow-up** | **Six-month follow-up** | ***P*-value** | **Three-week follow-up** | **Six-month follow-up** | ***P*-value** |
| Very easy | 0 (0.0%) | 1 (4.5%) | <0.001 | 0 (0.0%) | 1 (4.8%) | 0.017 |
| Easy | 1 (4.5%) | 1 (4.5%) | | 1 (4.8%) | 2 (9.5%) | |
| Normal | 5 (22.7%) | 10 (45.5%) | | 8 (38.1%) | 11 (52.4%) | |
| Difficult | 13 (59.1%) | 5 (22.7%) | | 12 (57.1%) | 6 (28.6%) | |
| Very difficult | 0 (0.0%) | 1 (4.5%) | | 0 (0.0%) | 0 (0.0%) | |
| Unknown | 3 (13.6%) | 4 (18.2%) | | 0 (0%) | 2 (9.5%) | |
| **Difficulties experienced (%)** | **Three-week follow-up** | **Six-month follow-up** | ***P*-value** | **Three-week follow-up** | **Six-month follow-up** | ***P*-value** |
| Expensive | 9 (%) | 4 (18.2%) | n/a | 6 (%) | 4 (19.0%) | n/a |
| Time consuming | 8 (%) | 6 (27.3%) | | 5 (%) | 7 (33.3%) | |
| Complex | 1 (%) | 4 (18.2%) | | 3 (0%) | 2 (9.5%) | |
| Little social support | 1 (%) | 1 (4.5%) | | 3 (%) | 2 (9.5%) | |
| Little positive effects | 2 (%) | 1 (4.5%) | | 0 (%) | 4 (19.0%) | |
| Other | 12 (57.1%) | 7 (31.8%) | | 12 (%) | 10 (47.6%) | |
| **How often were exceptions made (%)** | **Three-week follow-up** | **Three-month follow-up** | **Six-month follow-up** | **Three-week follow-up** | **Three-month follow-up** | **Six-month follow-up** |
| Every meal | 0 (0.0%) | 0 (0.0%) | 0 (0%) | 0 (0.0%) | 2 (9.5%) | 1 (4.8%) |
| One meal a day | 0 (0.0%) | 0 (0.0%) | 0 (0%) | 2 (9.5%) | 2 (9.5%) | 0 (0.0%) |
| One meal each 2–3 days | 3 (13.6%) | 1 (4.5%) | 4 (18.2%) | 8 (38.1%) | 3 (14.3%) | 9 (42.9%) |
| One meal a week | 4 (18.2%) | 6 (27.3%) | 5 (22.7%) | 3 (14.3%) | 9 (42.9%) | 7 (33.3%) |
| One meal every 2–3 weeks | 4 (18.2%) | 3 (13.6%) | 7 (31.8%) | 4 (19.1%) | 2 (9.5%) | 3 (14.3%) |
| One meal a month | 1 (4.5%) | 4 (18.2%) | 1 (4.5%) | 2 (9.5%) | 1 (4.8%) | 0 (0.0%) |
| Never | 7 (31.8%) | 5 (22.7%) | 0 (0%) | 2 (9.5%) | 2 (9.5%) | 0 (0.0%) |
| Unknown | 2 (9.1%) | 3 (13.6%) | 5 (22.7%) | 0 (0.0%) | 0 (0.0%) | 1 (4.8%) |
| **Exception meals (%)** | **Three-week follow-up** | **Three-month follow-up** | **Six-month follow-up** | **Three-week follow-up** | **Three-month follow-up** | **Six-month follow-up** |
| Main meal | 7 (31.8%) | 8 (36.4%) | 11 (50.0%) | 6 (28.6%) | 5 (23.8%) | 9 (42.9%) |
| Snacks | 2 (9.1%) | 5 (22.7%) | 1 (4.5%) | 6 (28.6%) | 4 (19.1%) | 3 (14.3%) |
| Both | 2 (9.1%) | 1 (4.5%) | 3 (13.6%) | 7 (33.3%) | 10 (47.6%) | 8 (38.1%) |
| Unknown | 11 (50.0%) | 8 (36.4%) | 7 (31.8%) | 2 (9.5%) | 2 (9.5%) | 1 (4.8%) |
| **Number of dropouts(%)[3]** | | | | | | |
| After 3 weeks | | 0 (0%) | | | 1 (4.8%) | |
| After 6 weeks | | 2 (9.1%) | | | 0 (0%) | |
| After 9 weeks | | 0 (0%) | | | 0 (0%) | |
| After 12 weeks | | 0 (0%) | | | 0 (0%) | |
| **Continuing the diet at the end of follow-up (%)** | | | | | | |
| Yes | | 17 (77.3%) | | | 15 (71.4%) | |
| Partially | | 1 (4.5%) | | | 2 (9.5%) | |
| No | | 1 (4.5%) | | | 4 (19.1%) | |
| Unknown | | 3 (13.6%) | | | 0 (0.0%) | |
| **Patient satisfaction (mean, SD)** | | **Low FODMAP diet N = 22** | | | **Endometriosis diet N = 21** | ***P*-value** |
| Taken serious by dietician | | 9.95 (0.23) | | | 9.95 (0.22) | n/a |
| Expertise dietician | | 9.63 (0.68) | | | 9.76 (0.54) | n/a |
| Shared decision making | | 9.42 (1.07) | | | 9.71 (0.56) | n/a |
| Negotiability of progress | | 9.37 (1.07) | | | 9.76 (0.54) | n/a |
| Information provision | | 9.47 (0.77) | | | 9.71 (0.56) | n/a |
| Accessibility dietician | | 9.79 (0.54) | | | 9.76 (0.63) | n/a |
| Self-reported effect of diet | | 9.26 (0.81) | | | 9.10 (0.89) | 0.667 |
| Recommend diet to others | | 9.37 (1.30) | | | 9.24 (1.00) | n/a |

1 FODMAP: fermentable oligo-, di-, mono-saccharides, and polyols.
2 Previous dietary guidelines such as eating vegetarian, vegan, halal, kosher gluten-, or lactose-free.
3 All women dropped out because of personal reasons such as moving to a foreign country or an illness in the family.
